# Use of the Smoking Cessation App Ex-Smokers iCoach and Associations With Smoking-Related Outcomes Over Time in a Large Sample of European Smokers: Retrospective Observational Study

**DOI:** 10.2196/45223

**Published:** 2023-08-22

**Authors:** Marthe BL Mansour, Wim B Busschers, Mathilde R Crone, Kristel M van Asselt, Henk C van Weert, Niels H Chavannes, Eline Meijer

**Affiliations:** 1 Department of General Practice Academic Medical Centre Amsterdam, Amsterdam University Medical Centres Amsterdam Netherlands; 2 Amsterdam Public Health Research Institute Amsterdam Netherlands; 3 Department of Public Health & Primary Care Leiden University Medical Centre Leiden Netherlands; 4 National eHealth Living Lab Leiden University Medical Center Leiden Netherlands

**Keywords:** smoking cessation app, digital smoking cessation intervention, user groups, trajectories of use patterns, engagement, smoking-related outcomes, smoker characteristics, European smokers, mobile phone

## Abstract

**Background:**

Digital interventions are increasingly used to support smoking cessation. Ex-smokers iCoach was a widely available app for smoking cessation used by 404,551 European smokers between June 15, 2011, and June 21, 2013. This provides a unique opportunity to investigate the uptake of a freely available digital smoking cessation intervention and its effects on smoking-related outcomes.

**Objective:**

We aimed to investigate whether there were distinct trajectories of iCoach use, examine which baseline characteristics were associated with user groups (based on the intensity of use), and assess if and how these groups were associated with smoking-related outcomes.

**Methods:**

Analyses were performed using data from iCoach users registered between June 15, 2011, and June 21, 2013. Smoking-related data were collected at baseline and every 3 months thereafter, with a maximum of 8 follow-ups. First, group-based modeling was applied to detect distinct trajectories of app use. This was performed in a subset of steady users who had completed at least 1 follow-up measurement. Second, ordinal logistic regression was used to assess the baseline characteristics that were associated with user group membership. Finally, generalized estimating equations were used to examine the association between the user groups and smoking status, quitting stage, and self-efficacy over time.

**Results:**

Of the 311,567 iCoach users, a subset of 26,785 (8.6%) steady iCoach users were identified and categorized into 4 distinct user groups: *low* (n=17,422, 65.04%), *mild* (n=4088, 15.26%), *moderate* (n=4415, 16.48%), and *intensive* (n=860, 3.21%) users. Older users and users who found it important to quit smoking had higher odds of more intensive app use, whereas men, employed users, heavy smokers, and users with higher self-efficacy scores had lower odds of more intensive app use. User groups were significantly associated with subsequent smoking status, quitting stage, and self-efficacy over time. For all groups, over time, the probability of being a smoker decreased, whereas the probability of being in an improved quitting stage increased, as did the self-efficacy to quit smoking. For all outcomes, the greatest change was observed between baseline and the first follow-up at 3 months. In the *intensive* user group, the greatest change was seen between baseline and the 9-month follow-up, with the observed change declining gradually in *moderate*, *mild*, and *low* users.

**Conclusions:**

In the subset of steady iCoach users, more intensive app use was associated with higher smoking cessation rates, increased quitting stage, and higher self-efficacy to quit smoking over time. These users seemed to benefit most from the app in the first 3 months of use. Women and older users were more likely to use the app more intensively. Additionally, users who found quitting difficult used the iCoach app more intensively and grew more confident in their ability to quit over time.

## Introduction

### Background

Europe has the world’s highest prevalence of smoking among adults. Despite a decrease in smoking among men and women, 26.3% of adults still use tobacco, as assessed in 2018 by the World Health Organization [1].

Over the last decade, internet access and use in Europe has increased steeply, with 92% of households in the European Union (EU) having access to the internet in 2021, compared with 71% in 2011 [2]. The proportion of individuals using a mobile phone for internet access has increased from 27% in 2012 to 75% in 2019 [3]. Digital smoking cessation interventions represent a relatively new approach to address tobacco use, comprising internet-, mobile phone–, and app-based interventions [4-6]. These interventions have the potential to reach a large number of individuals and are available at low costs, providing easy access at a time suited to the individual and advice tailored to specific needs [7].

Studies have shown that interactive, tailored internet-based interventions improve smoking cessation outcomes at 6-month follow-up similar to mobile phone–based interventions that use automated text messages [4,5]. Although some studies have shown a (small) positive influence of mobile apps on smoking-related outcomes, the evidence to support the effectiveness of these apps on smoking cessation outcomes remains inconsistent, mainly because of small sample sizes and heterogeneity of the interventions under study [6,8-15]. Moreover, not all apps are evidence based (using behavior change theories) or adhere to smoking cessation guidelines [16-20].

Ex-smokers iCoach (iCoach) was the most commonly used publicly available app for smoking cessation in Europe, with 404,551 unique users between 2011 and 2013, representing 0.34% of European smokers [21]. The app was launched by the European Commission as part of their campaign “Ex-smokers are unstoppable,” with the aim of enhancing motivation to quit and providing practical help for smoking cessation [21]. iCoach was freely accessible as a web-based platform and later as a mobile app via app stores, available in 23 different languages spoken in the 27 EU member states, and accessible to people of all ages. iCoach was assessed as a top-ranked app developed in the public sector [17] and was reviewed as interactive, addressing all the behavior change techniques (such as “advising on coping” or “rewarding abstinence”) used to evaluate various smoking cessation apps [22].

Web-based interventions aimed at prevention are often not used or used insufficiently, which is the reason for the often-observed small effect sizes [11,23]. The extent to which a digital intervention is used, such as smoking cessation apps, seems to be associated with behavioral outcomes [23-25]. Several studies have investigated use patterns for digital programs for smoking cessation using the number of app downloads, number of log-ins, or website use metrics [7,26,27]. Variables related to the use or trajectories of app use (such as gender, age, and a recent quit attempt) tend to vary across interventions [7,27,28].

A novel approach to analyzing the extent and patterns of apps for health behavior change is the analysis of latent trajectories of app use over time [28,29]. Little is known about engagement over time and the association between app use patterns and behavioral change outcomes. Goh et al [29] introduced the analysis of trajectory groups within app use of patients with type 2 diabetes mellitus with latent class growth modeling. More recently, Bricker et al [28] were the first to apply this methodology to analyze smoking cessation app data. Both studies were conducted in a trial setting [28,29]. Bricker et al [28] found that more intensive app use patterns were associated with higher odds of being abstinent from smoking at 12-month follow-up.

Analyzing user data of a real-world smoking cessation app could provide new insights into distinct use patterns over time of smokers who freely use such an app and provide useful information on both factors related to distinct use groups and the effect on smoking-related outcomes over time. This could support a more tailored design and dissemination of publicly available smoking cessation apps and eventually enhance user engagement and improved smoking-related outcomes. To achieve this, a large number of users are required, as many app users disengage.

Studying the efficacy of behavioral change apps should optimally be performed among those users who engage (ie, who use the app as intended) [30]. This differs from research on the adoption of such apps, which can be performed using a random sample to assess the engagement of the average user with the app.

### Objective

Using iCoach data from 311,567 users who participated between June 15, 2011 and June 21, 2013, we selected a subset of 26,785 (8.60%) participants who used the app for at least 3 months (≥1 follow-up measurement). This selection was made to analyze the efficacy of iCoach among users who had actually engaged. We investigated whether distinct patterns of intensity of iCoach use could be observed among these participants and whether the intensity of use was associated with the baseline characteristics of participants and smoking-related outcomes over time.

## Methods

### Study Design

This study used a longitudinal observational design. iCoach user data were obtained from users who were registered between June 15, 2011 and June 21, 2013.

The iCoach user data set was used to answer the following research questions: (1) *What are the distinct groups of users of iCoach app, based on duration and intensity of app use?* (2) *Which baseline characteristics are associated with user group membership?* and (3) *Is the intensity of app use associated with smoking-related outcomes over time?*

### Informed Consent and Data Privacy

Consent to use anonymized user data for research purposes was obtained upon registration. Upon registration, participants were asked to actively agree with the general terms of the app, including the privacy policy. On the basis of the country of the user, general terms were available in 1 of the 23 languages spoken in the EU member states. The privacy policy explained which data were collected, the purposes of data collection (eg, scientific research), and how users could have their data removed. This procedure satisfied the General Data Protection Regulation and Dutch Privacy Law requirements for consent. General terms in English are presented in Multimedia Appendix 1.

The study data were anonymous and untraceable to individual users. App users did not receive any compensation. The data were safely stored in a secured digital file on an internal drive with backup possibilities.

### Ethics Approval

The study was approved by the Medical Ethics Review Committee of the Leiden University Medical Centre, the Netherlands (reference:23-3061).

### iCoach App

iCoach was developed and hosted by BrandNewHealth for the campaign “Ex-smokers are unstoppable” run by the European Commission [21]. BrandNewHealth was a company that developed evidence-based digital health behavioral change interventions. iCoach was initially available as a web-based coaching platform, with the mobile app added later [21]. The iCoach was freely available in 23 languages spoken in 27 EU member states. Although the campaign was aimed at people aged 24 to 35 years living in Europe, iCoach was accessible to all people regardless of age.

iCoach combined elements of various behavior change theories, such as the Transtheoretical Model of Behavioral Change (TTM) [31], the Theory of Planned Behavior [32], and the Self-Determination theory [33]. In addition, elements of Cognitive Behavioral Therapy [34] were used. A positive approach was chosen, with a focus on the benefits of smoking cessation. A coaching platform offered a personalized program to help smokers quit, which consisted of the following elements.

**Figure 1:**
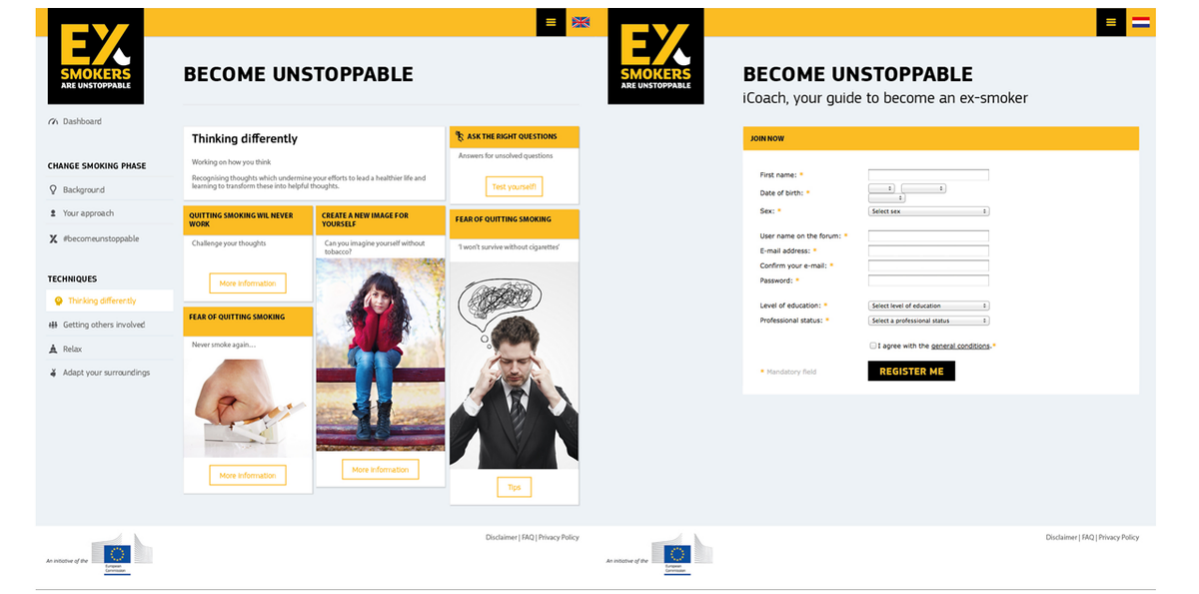
Screenshots of the Ex-smokers iCoach app.

Upon first log-in and every 3 months thereafter, users were requested to complete a Health Risk Assessment (HRA) questionnaire to assess smoking status and other smoking-related constructs. Questionnaire completion was mandatory to enable or continue the app use. Users were also asked to indicate their quitting stage based on the TTM [31]. Tailored information and advice on smoking cessation were offered based on the user’s quitting stage and additional questions on the user’s knowledge and motivation to quit smoking. Completing these additional questions was not compulsory, and they could be completed at any time. A diary provided an overview of current smoking behavior, with the option to choose a date to quit smoking. In addition, a library with information on smoking-related subjects and social support via a forum were available. Users received daily tips via email (these were based on their quitting stage and were activated by default, but the user could unsubscribe). After registration, users could access iCoach via both the website and mobile app. Screenshots of iCoach are provided in Figure 1.

### Study Participants

From the data of users who registered between June 15, 2011 and June 21, 2013, data of users younger than 18 years and of users with quitting stage 0 at baseline and error outliers were removed. This resulted in a data set comprising 311,567 iCoach users. Of these 311,567 iCoach users, a subset of 26,785 (8.6%) users was selected to answer our research questions. To obtain this subset, we first selected users who had completed the baseline HRA questionnaire and at least 1 follow-up HRA questionnaire. As the completion of the baseline HRA questionnaire was not mandatory, we selected those users who used the app for at least 80 days (instead of 89 days, which is the minimum number of days in 3 consecutive months) and completed the 3-month follow-up measurement. Without follow-up measurements, it would not have been possible to answer our research questions. In addition, app use for a certain amount of time would be necessary to investigate app use and smoking-related outcomes over time. Finally, for the choice of model for the app use trajectory analysis (see the *Statistical Analysis* section), we selected only those participants with a maximum app use rate of twice a day. The subset of 26,785 participants was defined as the steady iCoach users.

### Baseline and Follow-Up Measurements

#### Demographic Variables

After registration, the following data were collected: country (automatically collected from smartphone settings, 27 countries in total; Table S1 in Multimedia Appendix 2); date of birth; gender; education (primary school, secondary school, and higher education); professional category (employed, unemployed, retired, homemaker, student, and other); and registration date.

#### HRA Questionnaire

Individual users completed between 0 and 9 HRA questionnaires (with a maximum of 8 follow-up HRA measurements for the present data set, corresponding to approximately 24 months). Textbox 1 shows the information collected at each 3-month HRA.

From the data displayed in Textbox 1, we additionally computed the following variables: age; Heaviness of Smoking Index (HSI) [37]; smoking in the social environment (mean of the 3 measurements of smoking with family, colleagues, or friends); and Tobacco Control Scale (TCS; scores for each country based on the TCS 2013; higher TCS indicates stricter tobacco rules; see Table S1 in Multimedia Appendix 2 for the TCS per country) [38]. The professional category was regrouped into employed; unemployed; and all others (retired, homemaker, student, other).

**Data collected by Health Risk Assessment questionnaire.**

- Smoking status
  - Smoker or nonsmoker
- Quitting stage (based on the Transtheoretical Model [TTM] of Behavioral Change)
  - Stage 1 “I don’t want to quit smoking”
  - Stage 2 “I want to quit smoking but don’t know how or when”
  - Stage 3 “I plan to quit smoking in the near future”
  - Stage 4 “I recently quit smoking”
  - Stage 5 “I quit smoking more than 6 months ago”
  - Stage 0 “I have never smoked” [31]
- Days since last cigarette
- Number of cigarettes per weekday and per weekend day [35] from which the maximum number of cigarettes per day and per week was computed automatically [36]
- Time to first cigarette (TTFC) [35], that is, first cigarette after waking up
  - After 60 minutes
  - After 31 to 60 minutes
  - After 6 to 30 minutes
  - Within 5 minutes
- *To what extent are the people with whom you spend the most time smokers?*
  - Measured as 3 separate items for family, colleagues, or friends, using a Likert scale ranging from 0 (no one) to 6 (everyone)
- *How confident are you that you could resist smoking cigarettes?*
  - Self-efficacy measured using a Likert scale ranging from 0 (not confident) to 6 (very confident)
- *How motivated are you to stop smoking?*
  - Measured using a Likert scale ranging from 0 (not at all motivated) to 6 (strongly motivated)
- *How important is stopping smoking to you?*
  - Measured using a Likert scale ranging from 0 (not at all important) to 6 (very important)

#### App Use

We used the following variables to assess app use: first log-in date, last log-in date, duration of app use (time in days between the first and last log-in), and log-in counts (number of log-ins between 2 HRA measurements).

### Statistical Analysis

#### Overview

For the demographic and HRA variables collected at baseline, we computed means with SDs (continuous variables) and counts with percentages (categorical variables). We compared the distribution of categorical variables using Pearson chi-squared test and compared the means of continuous variables using a 2-tailed Student *t* test between the users selected for analysis of app use (steady users) and all other users.

#### Research Question 1

The app use rate was determined as follows. For each user, the sum of the total number of log-ins was averaged over the duration of app use in days. Group-based trajectory modeling using SAS Proc Traj (SAS Institute Inc), a statistical analysis software macro [39], was used to identify different groups (ie, latent groups) of iCoach steady users. This procedure required repeated count measurements over fixed time intervals. To this end, we assigned the individual log-in counts to these prespecified intervals assuming that the individual rate remained constant over the period of observation (of that individual user). We chose to model app use for approximately 1 year. To obtain regular intervals, we chose 6 periods of 60 days (6 × 60 = 360 days, which is approximately 1 year). For each user, this resulted in the assignment of their number of log-ins (based on their app use rate) over 6 fixed consecutive time periods of 60 days. The highly irregular patterns of individual app use limited our choice of models for use trajectory analysis. Therefore, we selected only those participants who used the app with a maximum rate of twice a day, and we considered them to be the steady users. As described under the *Study Participants* section, this resulted in a final subset of 26,785 steady users. This selection cannot be considered a random sample of all app users, and our findings are restricted to these “steady” users. The analysis revealed 4 distinct groups where the zero-inflated Poisson model for the observed counts provided the most stable results. Within the 4 group models, the final model was selected based on the smallest Bayesian Information Criterion. Users were classified into the user group for which they had the highest probability of being a member. Table S2 in Multimedia Appendix 2 shows the variability in the rate of app use for all iCoach users according to the duration of app use. Due to this high variability in rates, many potentially interesting models did not converge, and we restricted our analyses to individuals with app use rates less than 2 per day to obtain reliable results.

#### Research Question 2

**Figure 2:**
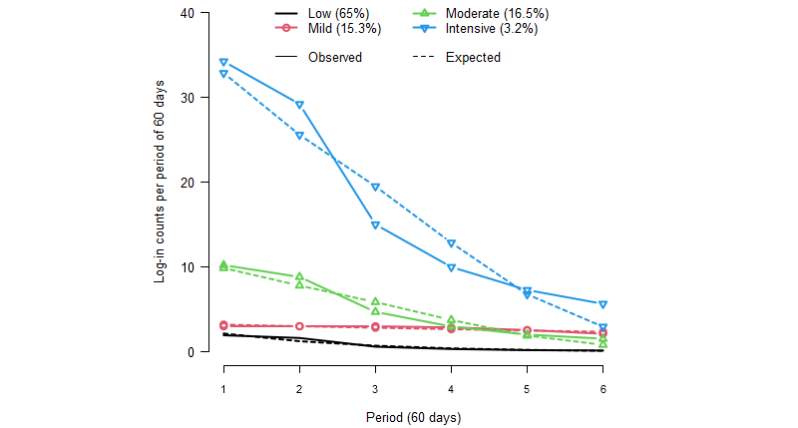
iCoach user groups zero-inflated Poisson model.

The results of the group analyses provided an ordinal classification of app use. This analysis was performed using the area under the curve (Figure 2). An ordinal logistic regression was used to assess which baseline demographic characteristics were associated with user group membership and included those treated as categorical variables, namely, gender, education, and professional category, and those treated as continuous variables, namely, age, HSI, self-efficacy, importance of quitting, and smoking in the social environment. Motivation to stop was not included, as this variable correlated strongly with self-efficacy. Variables were selected using backward selection, with a removal probability of 0.2. The assumption of proportional odds was investigated using the parallel lines test of Peterson and Harrell [40] and graphical analysis, which showed that the proportional odds were partially met. In addition, multinomial logistic regression was performed to investigate the associations between the baseline variables and the different groups, with more detail and without the proportional odds assumption, using the same independent variables as in the ordinal model. The results of the multinomial logistic regression are presented in Multimedia Appendix 2. SPSS Statistics (version 28.0; IBM Corp) was used for the analysis.

#### Research Question 3

Generalized estimating equations (GEEs) were used to explore the association between user groups and smoking status (binary, ie, nonsmoker=0 and smoker=1), quitting stage, and self-efficacy (continuous) over time. Subsequently, a Wald multiple degree of freedom test of association was used to investigate whether user groups evolved differently over time. Patterns of informative missingness were observed after the fourth HRA measurement; if the measurement of an outcome variable was missing, it was also missing in all subsequent HRA measurements. Therefore, we used the first 4 HRA measurements at baseline and at 3, 6, and 9 months. For each user group, an estimate was computed for each time point. For the binary outcome (smoking status), estimates were produced on the logit scale, whereas for the continuous outcomes, estimates were produced on the original Likert scale. To investigate the changes over time, the models contained a time variable, a user group variable, and an interaction term between them. Potential confounders of age, gender, and education were also included in the models. The largest user group with the lowest use was chosen as the reference group. For interpretation purposes, predicted probabilities were calculated for smoking status and predicted means for quitting stage and self-efficacy. Analyses were performed using R (version 4.0.3; R Foundation for Statistical Computing) with R package *geepack* [41,42].

## Results

### Baseline Characteristics of iCoach Users

Of the total 311,567 iCoach users, the demographic and baseline variables for the subset of 26,785 (8.6%) steady iCoach users with ≥1 follow-up measurement who were included in the app use analysis are presented in Table 1.

In the subset of steady users (n=26,785), the mean age was 36.5 (SD 11.4) years, 12,695 (47.4%) were women, 16,106 (60.13%) had completed higher education, and 17,743 (66.24%) were employed. There were 22,027 (N=26,785, 82.24%) current smokers, of whom 10,416 (47.29%) were in stage 3 (“I plan to quit smoking in the near future”). Users smoked an average of 16.5 (SD 8.3) cigarettes per day, and most users (13,938/26,785, 52.04%) had a medium HSI. The mean motivation to stop score was 4.1 (SD 1.6), and the mean self-efficacy score was 2.9 (SD 1.9).

Table S3 in Multimedia Appendix 2 provides a separate overview of the baseline variables for all 311,567 iCoach users.

**Table 1:** Baseline characteristics of iCoach user groups.

Characteristic			Low^a^ (n=17,422)		Mild^a^ (n=4088)		Moderate^a^ (n=4415)		Intensive^a^ (n=860)		All user groups (n=26,785)
Age (years)^b^, mean (SD)			35.2 (10.9)		37.0 (11.3)		39.5 (11.9)		43.9 (12.6)		36.5 (11.4)
Women, n (%)			7829 (44.94)		2050 (50.15)		2397 (54.29)		419 (48.72)		12,695 (47.4)
**Education, n (%)**											
	Primary education	593 (3.4)		106 (2.59)		135 (3.06)		43 (5)		877 (3.27)	
	Secondary education	6255 (35.9)		1499 (36.67)		1677 (37.89)		371 (43.14)		9802 (36.6)	
	Higher education	10,574 (60.74)		2483 (60.74)		2603 (58.96)		446 (51.86)		16,106 (60.13)	
**Professional category, n (%)**											
	Employed	11,600 (66.58)		2773 (67.83)		2874 (65.1)		496 (57.67)		17,743 (66.24)	
	Unemployed	1339 (7.69)		289 (7.07)		314 (7.11)		62 (7.21)		2004 (7.48)	
	All other^c^	4483 (25.73)		1026 (25.1)		1227 (27.79)		302 (35.12)		7038 (26.28)	
**Country^d^, n (%)**											
	Belgium	1137 (6.53)		389 (9.52)		494 (11.18)		131 (15.23)		2151 (8.03)	
	France	992 (5.69)		255 (6.24)		282 (6.39)		55 (6.4)		1584 (5.91)	
	Germany	785 (4.51)		190 (4.65)		256 (5.8)		54 (6.28)		1285 (4.8)	
	Hungary	898 (5.15)		242 (5.92)		230 (5.21)		49 (5.7)		1419 (5.3)	
	Italy	1277 (7.33)		389 (9.52)		419 (9.49)		76 (8.84)		2161 (8.07)	
	Netherlands	679 (3.9)		225 (5.5)		198 (4.48)		36 (4.19)		1138 (4.25)	
	Poland	849 (4.87)		186 (4.55)		201 (4.55)		39 (4.53)		1275 (4.76)	
	Portugal	948 (5.44)		248 (6.07)		250 (5.66)		42 (4.88)		1488 (5.56)	
	Romania	833 (4.78)		168 (4.11)		153 (3.47)		33 (3.84)		1187 (4.43)	
	Spain	2023 (11.61)		490 (11.99)		541 (12.25)		103 (11.98)		3157 (11.79)	
	United Kingdom	3104 (17.82)		345 (8.43)		452 (10.24)		77 (8.95)		3978 (14.85)	
TCS^e^, mean (SD)^f^			51.0 (13)		48.4 (10.8)		48.7 (11.3)		48.2 (10.9)		50.1 (12.4)
Smoking, n (%)			14,364 (82.45)		3379 (82.66)		3607 (81.7)		677 (78.72)		22,027 (82.24)
**Quitting stage, n (%)**											
	Stage 1	275 (1.58)		91 (2.22)		85 (1.93)		16 (1.86)		467 (1.74)	
	Stage 2	7138 (40.97)		1748 (42.76)		1881 (42.6)		377 (43.84)		11,144 (41.61)	
	Stage 3	6951 (39.9)		1540 (37.67)		1641 (37.19)		284 (33.02)		10,416 (38.89)	
	Stage 4	2617 (15.02)		672 (16.44)		785 (17.78)		164 (19.07)		4238 (15.82)	
	Stage 5	441 (2.53)		37 (0.91)		23 (0.52)		19 (2.21)		520 (1.94)	
Cigarettes per day^g^, mean (SD)			16.5 (8.3)		16.4 (8.3)		16.3 (8.3)		17.2 (9)		16.5 (8.3)
**HSI^h,i^, n (%)**											
	Low (0-1)	3.459 (19.85)		865 (21.16)		920 (20.84)		154 (17.91)		5398 (20.15)	
	Medium (2-4)	9199 (52.8)		2083 (50.95)		2232 (50.55)		424 (49.3)		13,938 (52.04)	
	High (5-6)	1.774 (10.18)		433 (10.59)		464 (10.51)		100 (11.63)		2771 (10.35)	
Motivation to stop, mean (SD)			4.1 (1.6)		4.0 (1.6)		4.1 (1.6)		4.1 (1.7)		4.1 (1.6)
Self-efficacy, mean (SD)			3.0 (1.9)		2.8 (1.9)		2.8 (1.9)		2.7 (2)		2.9 (1.9)
Importance of quitting, mean (SD)			5.2 (1.2)		5.3 (1.1)		5.4 (1.1)		5.3 (1.2)		5.3 (1.2)
Smoking in social environment, mean (SD)			2.6 (1.4)		2.4 (1.3)		2.3 (1.3)		2.1 (1.3)		2.5 (1.4)

^a^Labels of the groups, following the ordinal ordering with low (least-intensive app use) to intensive (most-intensive app use).

^b^Range 18 to 85 years.

^c^All other include retired, homemaker, student, and other.

^d^Listed countries with frequencies >4% of the overall sample; see Multimedia Appendix 2 for the frequencies of all 27 countries.

^e^TCS: Tobacco Control Scale.

^f^Range 31 to 74.

^g^Range 0 to 40; a total of >40 cigarettes per day was computed as 40 cigarettes per day.

^h^Percentage of all users in each group; the percentage of users missing overall was 17.5%.

^i^HSI: Heaviness of Smoking Index.

### Research Question 1—App Use

There were 4 distinct iCoach user groups among participants who had used the app for ≥80 days, with a rate of <2 log-ins per day (n=26,785 users). Figure 2 illustrates the 4 groups, presented in an ordinal manner based on the intensity of app use: *low* (17,422/26,785, 65.04% of users); *mild* (4088/26,785, 15.26% of users); *moderate* (4415/26,785, 16.48% of users); and *intensive* (860/26,785, 3.21% of users). Descriptive statistics for the number of log-ins for each group per period of use are presented in Table S4 in Multimedia Appendix 2.

*Low* users started with around 2 log-ins in the first period of 60 days, declining to 1 log-in between 120 and 180 days and to (nearly) no log-ins from the third period onward (180-360 days). *Mild* users started with 3 log-ins in the first period of 60 days, and their use remained constant up to the fourth period (240 days), followed by a slow decline in use to <3 log-ins up to the sixth period (360 days). *Moderate* users started with 10 log-ins in the first period of 60 days, going to 9 log-ins between the first and second period (60-120 days), followed by a drop in use to 4 log-ins between 120 and 180 days and a more gradual decrease to <3 log-ins after 180 days. *Intensive* users started with 34 log-ins in the first period, which declined to 29 log-ins in the second period, followed by a steep decline to 15 log-ins in the third period and a further drop to 10 log-ins in the fourth period (180-240 days), after which the use gradually decreased to 6 log-ins per period.

*Low* and *mild* users had a more constant pattern over time, whereas *moderate* and *intensive* users had more intensive use at the start, which decreased steeply over time.

### Research Question 2—Baseline Characteristics Associated With User Group Membership

Table 2 presents the results of the ordinal logistic regression of the characteristics associated with user group membership. Users with a 1-point increase in age or a 1-point increase on the Likert scale scoring the individuals’ perception of importance of quitting had higher odds of being intensive app users, with an odds ratio (OR) of 1.03 (95% CI 1.03-1.03; *P*<.001) for age and an OR of 1.04 (95% CI 1.01-1.06; *P*=.004) for importance of quitting. Men (compared with women) were less likely to use the app intensively (OR 0.78, 95% CI 0.74-0.82; *P*<.001), as were employed users compared with the “all other” employment category (OR 0.89, 95% CI 0.84-0.95; *P*=.001). Furthermore, users with a 1-point increase in his were less likely to use the app intensively (OR 0.94, 95% CI 0.93-0.96; *P*<.001), as were users with a 1-point increase in self-efficacy (OR 0.97, 95% CI 0.96-0.99; *P*=.001), users with a 1-point increase in smoking in their social environment (OR 0.92, 95% CI 0.90-0.94; *P*<.001), and users with a 1-point increase in the TCS of the user’s country (OR 0.98, 95% CI 0.98-0.99; *P*<.001). Education was not significantly associated with group membership (all *P*>.05).

The results from the multinomial logistic regression (with the *low* users as the reference group) provided more detailed information on the association of characteristics with the different trajectory groups (Table S5 in Multimedia Appendix 2).

**Table 2:** Ordinal logistic regression results of characteristics associated with iCoach user group membership^a^.

Characteristic			OR^b^ (95% CI)		*P* value
Age			1.03 (1.03-1.03)		<.001
Gender^c^			0.78 (0.74-0.82)		<.001
**Education^d^**					
	Primary school	0.87 (0.74-1.01)		.07	
	Secondary school	1.03 (0.97-1.09)		.29	
**Professional category^e^**					
	Employed	0.89 (0.84-0.95)		.001	
	Unemployed	0.91 (0.82-1.02)		.12	
HSI^f^			0.94 (0.93-0.96)		<.001
Self-efficacy			0.97 (0.96-0.99)		.001
Importance of quitting			1.04 (1.01-1.06)		.004
Smoking in social environment			0.92 (0.90-0.94)		<.001
TCS^g^			0.98 (0.98-0.99)		<.001

^a^For this analysis, an ordinal classification of user groups based on the intensity of app use was used: low to intensive.

^b^OR: odds ratio.

^c^For the gender category, “women” are the reference group.

^d^For the education category, “higher education” is the reference group.

^e^For the professional category, the category “all other” is the reference group (including retired, homemaker, student, and other).

^f^HSI: Heaviness of Smoking Index.

^g^TCS: Tobacco Control Scale.

### Research Question 3—Association Between User Group and Smoking-Related Outcomes

The Wald multiple degree of freedom test of association applied to the GEE models showed evidence that smoking status (*χ*^2^_9_=23.1, *P*=.006), quitting stage (*χ*^2^_9_=20.4, *P*=.02), and self-efficacy (*χ*^2^_9_=40.3, *P*<.001) evolved differently over time in the 4 user groups. The GEE analyses showed that all 4 user groups were significantly associated with subsequent smoking status, quitting stage, and self-efficacy but did not behave similarly over time. Tables 3 to 5 present the predicted probabilities for smoking status and the predicted means for quitting stage and self-efficacy, respectively. Tables S6-S8 in Multimedia Appendix 2 provide an overview of the GEEs for each outcome, including *P* values.

The probability that a user would still be a smoker tended to decrease over time (Table 3) with a nonlinear trend. For all user groups, the steepest decline in probability was observed between baseline and 3 months (compared with the referent), with only a slight decrease over subsequent measurements. *Intensive* users showed the largest decline between baseline and 9 months (compared with the referent; *P*=.049; Table S6 in Multimedia Appendix 2). *Moderate* users showed a higher probability of being a smoker at baseline and significant changes in the probability of being a smoker between all follow-up measurements (compared with the referent; *P* values of the GEE for moderate users for follow-up measurements at 3, 6, and 9 months were <.001, .003, and .01, respectively; Table S6 in Multimedia Appendix 2).

Similarly, the means of the quitting stage (ie, the higher quitting stage) tended to increase over time (Table 4). For all user groups, the steepest increase in the intention to quit was observed between baseline and 3 months (compared with the referent). Furthermore, *intensive* users showed the largest increase in mean between baseline and 9 months (compared with the referent; *P*=.009; Table S7 in Multimedia Appendix 2). *Moderate* users showed the next largest increase in means (compared with the referent), with the lowest mean for the quitting stage at baseline and significant changes between all follow-up measurements (compared with the referent; *P* values of the GEE for moderate users for follow-up measurements at 3, 6, and 9 months were .003, <.001, and .003, respectively; Table S7 in Multimedia Appendix 2).

A comparable trend was observed for self-efficacy in smoking cessation over time. For all user groups, the steepest increase in self-efficacy was observed between baseline and 3 months (compared with the referent). Yet again, *intensive* users showed the largest increase in mean self-efficacy score between baseline and 9 months (compared with the referent; *P*=.004; Table S8 in Multimedia Appendix 2), followed by *moderate, mild,* and *low* users (*P* values of the GEE for moderate, mild, and low users at 9 months were <.001, .009, and <.001, respectively; Table S8 in Multimedia Appendix 2).

**Table 3:** Model-predicted probabilities of smoking status by iCoach user groups over time^a^.

Time points	Low		Mild		Moderate		Intensive	
	PP^b^ (95% CI)	SE	PP (95% CI)	SE	PP (95% CI)	SE	PP (95% CI)	SE
Baseline^c^	0.799 (0.730-0.854)	0.032	0.858 (0.797-0.889)	0.023	0.845 (0.788-0.889)	0.026	0.797 (0.710-0.863)	0.039
3 months^d^	0.546 (0.452-0.637)	0.048	0.561 (0.474-0.644)	0.044	0.488 (0.398-0.578)	0.046	0.499 (0.391-0.608)	0.056
6 months^e^	0.494 (0.400-0.588)	0.048	0.500 (0.414-0.586)	0.044	0.460 (0.372-0.550)	0.046	0.430 (0.327-0.540)	0.055
9 months^f^	0.475 (0.382-0.569)	0.048	0.474 (0.389-0.561)	0.044	0.456 (0.368-0.546)	0.046	0.360 (0.264-0.468)	0.053

^a^Predicted probability is shown for being a smoker.

^b^PP: predicted probability.

^c^Baseline Health Risk Assessment measurement.

^d^First follow-up Health Risk Assessment measurement at 3 months.

^e^Health Risk Assessment measurement at 6-month follow-up.

^f^Health Risk Assessment measurement at 9-month follow-up.

**Table 4:** Model-predicted means of quitting stage by iCoach user groups over time^a^.

Time points	Low		Mild		Moderate			Intensive	
	PM^b^ (95% CI)	SE	PM (95% CI)	SE	PM (95% CI)	SE	PM (95% CI)		SE
Baseline^c^	2.81 (2.63-2.98)	0.0886	2.63 (2.47-2.79)	0.0822	2.62 (2.46-2.79)	0.0844	2.72 (2.52-2.92)		0.1028
3 months^d^	3.32 (3.15-2.50)	0.0906	3.24 (3.08-3.40)	0.0824	3.30 (3.13-3.47)	0.0856	3.30 (3.10-3.50)		0.1023
6 months^e^	3.56 (3.38-3.74)	0.0931	3.49 (3.33-3.66)	0.0834	3.60 (3.42-3.77)	0.0889	3.56 (3.34-3.77)		0.1106
9 months^f^	3.70 (3.51-3.89)	0.0950	3.66 (3.50-3.82)	0.0839	3.72 (3.55-3.90)	0.0903	3.89 (3.67-4.11)		0.1134

^a^Predicted mean is shown for quitting stage on the original Likert scale.

^b^PM: predicted mean.

^c^Baseline Health Risk Assessment measurement.

^d^First follow-up Health Risk Assessment measurement at 3 months.

^e^Health Risk Assessment measurement at 6-month follow-up.

^f^Health Risk Assessment measurement at 9-month follow-up.

**Table 5:** Model-predicted means of self-efficacy by iCoach user groups over time^a^.

Time points	Low		Mild		Moderate		Intensive	
	PM^b^ (95% CI)	SE	PM (95% CI)	SE	PM (95% CI)	SE	PM (95% CI)	SE
Baseline^c^	3.11 (2.77-3.45)	0.172	2.77 (2.47-3.07)	0.154	2.84 (2.52-3.16)	0.163	2.81 (2.41-3.21)	0.204
3 months^d^	3.74 (3.41-4.07)	0.169	3.67 (3.37-3.97)	0.153	4.05 (3.73-4.37)	0.163	4.20 (3.82-4.57)	0.193
6 months^e^	4.04 (3.71-4.37)	0.169	3.95 (3.65-4.25)	0.153	4.19 (3.87-4.51)	0.163	4.27 (3.89-4.65)	0.194
9 months^f^	4.18 (3.85-4.51)	0.169	4.11 (3.81-4.41)	0.154	4.37 (4.05-4.69)	0.162	4.39 (4.02-4.77)	0.192

^a^Predicted mean is shown for self-efficacy on the original Likert scale. Adjusted for age (36.5 years), gender (man), and education (primary education).

^b^PM: predicted mean.

^c^Baseline Health Risk Assessment measurement.

^d^First follow-up Health Risk Assessment measurement at 3 months.

^e^Health Risk Assessment measurement at 6-month follow-up.

^f^Health Risk Assessment measurement at 9-month follow-up.

## Discussion

### Principal Findings

iCoach provided a real-world data set with the largest number of individual users of a smoking cessation app currently available. For this study, the demographic and baseline variables of 26,785 iCoach steady users were presented, with a relatively long follow-up period of approximately 24 months. Among these iCoach steady users (n=26,785), a total of 4 distinct user groups were identified with differences in the intensity of app use over time: *low* (n=17,422, 65.04%), *mild* (n=4088, 15.26%), *moderate* (n=4415, 16.48%), and *intensive* (n=860, 3.21%) users.

Older age groups and increased Likert scale scores on the importance of quitting smoking were associated with higher odds of more intensive app use, whereas men, employed individuals, heavy smokers, and those having higher self-efficacy to quit smoking were associated with lower odds of more intensive app use.

The user groups were significantly associated with subsequent smoking status, quitting stage, and self-efficacy over time. For all groups, users were less likely to be smokers and more likely to have an improved quitting stage and increased self-efficacy over time. The greatest changes in these outcomes (predicted probabilities for smoking status, predicted means for quitting stage, and self-efficacy) were observed between baseline and 3 months. *Intensive* users showed the greatest change between baseline and 9-month follow-up in terms of chances of being a smoker, improved quitting stage, and improved self-efficacy. This was accompanied by a gradual decline in the degree of these observed changes in *moderate*, *mild*, and *low* users.

### Strengths and Limitations

The iCoach data provided novel insights into real-world app use, specifically into the app use patterns of steady users and the association of use with smoking-related outcomes over time. Nonadherence and discontinuation of digital health interventions can lead to small sample sizes, which makes it difficult to detect user patterns or the effect of app use on smoking or health-related outcomes [11,23,43]. The large data set used in this study provided a unique opportunity to analyze use over a longer follow-up period. In addition, it enabled the detection of smaller differences in characteristics associated with user group membership and smoking-related outcomes. Our analysis was limited by the fact that measurements were taken every 3 months instead of more frequently. Therefore, the potential short-term effects of app use on smoking-related outcomes could have been overlooked. All outcomes were self-reported and there was no biochemical validation of the smoking status outcome. Although data were not collected in a controlled trial setting, previous studies of relatively low-demand smoking cessation interventions showed few discrepancies between self-reported and biochemically validated abstinence [44,45]. In addition, organizing biochemical validation for such a large number of users would be extremely challenging and could result in a restricted number of participants or a selection bias, with users who quit smoking being more likely to participate in biochemical validation. iCoach users mentioned the obligatory completion of lengthy questionnaires as a drawback [21], which may have resulted in the discontinuation of app use. Another important limitation is that it was unknown if and to what extent users simultaneously used other smoking cessation aids or supports (eg, from a clinician). For the modeling of app use and analysis of the distinct user groups, we used the user’s personal use based on the number of log-ins over fixed periods of 60 days. Modeling app use was necessary to identify distinct user groups. As modeling data differ from actual observations, the results should be interpreted as trends rather than causal relationships. Moreover, the characteristics associated with group membership were based on a subset of steady users who had used the app for ≥80 days (8.60% of all users). This selection was required to obtain groups of users who completed at least 1 follow-up measurement and yield a stable model. Because the analysis was based on complete cases, this could have led to an overestimation of the association between the use groups and baseline characteristics or smoking-related outcomes. Finally, the subset of 8.60% of all iCoach users was not a random sample; therefore, the results do not represent the app use of all iCoach users. Many iCoach users used the app for 0 or 1 day after installation, which raises questions about them being actual “users.” In addition, the results cannot be generalized to average users of publicly available smoking cessation apps. This study aimed to examine the efficacy of the app and not its adoption. Therefore, we did not draw a random sample, as we wanted to study the efficacy of the app among those who engaged. The fact that only a small subset of users of a widely disseminated app engaged for a longer period could be illustrative of the actual engagement of publicly available smoking cessation apps.

### Comparison With Prior Work

Broadly, the 4 user groups used the app most intensively during the first 3 periods, which corresponds to the first 180 days of app use. *Low* and *mild* users had a more constant pattern with fewer log-ins, whereas *moderate* and *intensive* users began with more log-ins but showed a steep decline over time. In comparison, the few other studies that used a similar analysis for smoking cessation or health promotion apps (all performed in trial settings) studied short-term use of 1 to several weeks, with only a few users using the intervention for a longer period [7,26-29,46]. The 26-week iCanQuit users of Bricker et al [28] could be considered similar to our *intensive* users, with app users starting at a higher log-in rate with continuation of app use over at least several months. Looking into whether the 4 different groups of users make sense, we considered the *low* and *mild* users to differ from one another as *low* users practically stopped using the app after 180 days, whereas *mild* users continued to use the app, albeit on a limited level. The large sample size enabled the detection of a relatively small group of *intensive* users. We consider this smaller group to be informative, as its pattern differs from those of the other groups [47,48], and its association with smoking-related outcomes was stronger.

Our study shows different patterns of use of steady users of a publicly available app, with users showing either a more constant but lower log-in rate or initial higher log-ins but with a steeper decline over time. Analysis of another publicly available digital smoking cessation intervention suggested that despite most users not using the app or stopping its use prematurely, there is a subgroup of users who find the intervention beneficial and continue to use it [7,49]. In this study, app use was studied in a subset of iCoach users who completed ≥1 follow-up measurements and used the app for at least 80 days. This was not a random sample; therefore, the conclusions are only applicable to this specific subset. However, we consider this to be a proper reflection of the performance of iCoach. As mentioned by Eysenbach [50], high dropout rates seem to be a natural feature of self-help apps, and it is therefore important to distinguish between users who immediately drop out and those who slowly taper off the use. There were differences in the baseline characteristics of all iCoach users and the subset of users with ≥1 follow-up measurements. For example, although more men than women initially subscribed to the app, more women actually used it. At baseline, steady iCoach users were more motivated to stop smoking and considered quitting to be more important, compared with the other iCoach users. Although there was an incentive for all users to install the app at baseline, a higher motivation could have played a role in actual engagement with iCoach [51]. It could be that users with higher log-in numbers are more motivated to use the app; however, if this only happens over a short period (eg, 1 week), the contribution of the app to the quit attempt could be questioned. Our results suggest that among the steady users who engaged with the app for at least 80 days and with a log-in rate of maximally twice a day, patterns of more intensive app use are associated with better smoking-related outcomes. In our analysis, we could not include the users who used the app for at least 80 days with a use rate of >2 log-ins per day. A future study (without app use trajectory analysis) on these users could provide information on their characteristics and how they perform on smoking-related outcomes.

Of the relatively few studies performed, female individuals and (increasing) age were often identified as factors associated with more intensive use of digital smoking cessation [7,27,28,52] or other digital behavior change interventions [30]. With a mean age of 36.5 years, the user groups might have benefited most from the iCoach. However, with rapid digitalization among younger generations [53], younger adults and men might similarly benefit from these types of interventions in the future. Perski et al [30] found that higher education was also associated with increased intervention use, but we did not, suggesting that users benefited from iCoach regardless of their educational level. However, it is important to note that the educational level was higher in the subset of users analyzed in this study than among all iCoach users (60.13% vs 49.49%, respectively). Evaluation of the *StopCoach* app showed that users who received professional support to quit smoking used the app for longer periods (and were more inclined to quit smoking) [46]. For iCoach users, it was not known whether they simultaneously used professional support to quit smoking.

Intensive iCoach app use was associated with higher smoking cessation rates, quitting stage, and self-efficacy over time. Our findings suggest that, in the subset of steady users, iCoach stimulated users to stop smoking and increased their confidence in being able to quit. The extent of app use seems to be associated with the effectiveness of digital interventions [25]. Indeed, in our study, we found that more intensive app users experienced greater changes in smoking-related outcomes. In recent studies by Bricker et al [27,28], long-term users of smoking cessation apps had higher odds of being abstinent at 12-month follow-up, compared with short-term users. Similarly, in our study, more intensive users showed a larger decline in the predicted probability of being a smoker over time. Interestingly, the largest change in the predicted probability was observed between the baseline and 3-month follow-up. Thereafter, there were only minor additional changes in the predicted probability, with an overlap of the CIs of subsequent follow-up measurements. This could imply that at the population level, the greatest benefit of smoking cessation apps is obtained in the first months of app use. On the basis of our results, it is not possible to know if individual users resumed smoking after 3-month follow-up, but in such a situation, the app could potentially be beneficial, as many smokers perform about 6 quit attempts on average before successful long-term abstinence is achieved [54,55].

iCoach used the TTM to assess changes in the quitting stage. It is important to note that smokers do not go through the stages in a linear way and can, for example, jump one stage or suddenly stop smoking without previous plans [56]. However, instead of reflecting precise changes in the quitting stage, as used in our study with a large sample, it reflects a trend of increased willingness to quit smoking or maintain abstinence.

Self-efficacy is an important predictor of successful quit attempts [57,58]. We did not perform a mediation analysis; however, it is possible that the increase in self-efficacy (partially) played a role in the decrease in the predicted probability of being a smoker over time. We observed not only that users with higher self-efficacy were less inclined to use iCoach intensively but also that more intensive app use was associated with a higher predicted mean self-efficacy score over time. This could imply that those who found quitting difficult used the iCoach app more intensively and grew more confident in their ability to quit as their self-efficacy improved over time.

As significant changes in the outcomes of all user groups were observed, it could be argued that these changes were due to the natural course of smoking behavior over time, as it was not possible to compare the subset with the total group of users. However, more intensive use was associated with greater improvements in the outcomes. Nevertheless, the association between use and smoking-related outcomes should be interpreted as a trend rather than a causal relationship. A randomized trial could not address the issue of causality between app use and smoking-related outcomes, as it is not possible to randomize users to use groups.

### Conclusions

This real-world study evaluated a large-scale smoking cessation app. In the engaged subset of users, more intensive app use was associated with higher smoking cessation rates, increased quitting stage, and higher self-efficacy to quit smoking over time. Users seemed to benefit most from the app in the first 3 months of use. Women and relatively older users were more likely to use the app more intensively. Users who found quitting difficult used the iCoach app more intensively and grew more confident in their ability to quit over time.

## Appendix

Multimedia Appendix 1 General terms.

Multimedia Appendix 2 Supplementary data tables.

## Abbreviations

EU: European Union
GEE: generalized estimating equation
HRA: Health Risk Assessment
HSI: Heaviness of Smoking Index
OR: odds ratio
TCS: Tobacco Control Scale
TTM: Transtheoretical Model of Behavioral Change

## Data Availability

The data sets analyzed during this study are not publicly available because users did not provide consent for the public availability of collected data but are available from the corresponding author upon reasonable request.
