# Supplementary material for: Use of the Smoking Cessation App Ex-Smokers iCoach and Associations With Smoking-Related Outcomes Over Time in a Large Sample of European Smokers: Retrospective Observational Study
Source: J Med Internet Res. 2023 Aug 22;25:e45223. doi: 10.2196/45223 (PMC10481207; doi:10.2196/45223)
Supplement: Multimedia Appendix 1 [file jmir_v25i1e45223_app1.docx]

## MULTIMEDIA APPENDIX 1

**General Terms ‘Exsmokers iCoach’**

1. General.

The iCoach website and iCoach mobile applications are managed by Brand New Day NV (BrandNewDay), with registered offices at Park 51 Site, Persilstraat 51C, bus 301, in 3020 Herent, Belgium, entered in the RLP Leuven, company number 0480.270.358. These general terms and conditions apply to [http://www.stopsmokingcoach.eu](https://eur04.safelinks.protection.outlook.com/?url=http%3A%2F%2Fwww.stopsmokingcoach.eu%2F&data=05%7C01%7Cm.b.mansour%40amsterdamumc.nl%7C608cb3aa630e4debfb2b08db68db17c5%7C68dfab1a11bb4cc6beb528d756984fb6%7C0%7C0%7C638219062426694890%7CUnknown%7CTWFpbGZsb3d8eyJWIjoiMC4wLjAwMDAiLCJQIjoiV2luMzIiLCJBTiI6Ik1haWwiLCJXVCI6Mn0%3D%7C3000%7C%7C%7C&sdata=ECO%2FLo6zmuBLW%2FlfDC%2BTgABbEtoQyR82JDNsVcr6YzA%3D&reserved=0), the iCoach mobile applications and to all information, including e-mails, sent from [http://www.stopsmokingcoach.eu](https://eur04.safelinks.protection.outlook.com/?url=http%3A%2F%2Fwww.stopsmokingcoach.eu%2F&data=05%7C01%7Cm.b.mansour%40amsterdamumc.nl%7C608cb3aa630e4debfb2b08db68db17c5%7C68dfab1a11bb4cc6beb528d756984fb6%7C0%7C0%7C638219062426694890%7CUnknown%7CTWFpbGZsb3d8eyJWIjoiMC4wLjAwMDAiLCJQIjoiV2luMzIiLCJBTiI6Ik1haWwiLCJXVCI6Mn0%3D%7C3000%7C%7C%7C&sdata=ECO%2FLo6zmuBLW%2FlfDC%2BTgABbEtoQyR82JDNsVcr6YzA%3D&reserved=0) ("website"), and notifications, sent from the iCoach mobile applications ("app"). By accessing and using the website and app, you undertake to accept and abide by these terms and conditions.

2. Access to and use of the website and app

Each time you access and use the website and app you undertake to: - act in good faith and with reasonable caution and not use the website or app unlawfully; - not use the website or app for the transmission or posting of viruses or illegal or unlawful material or inappropriate material (including libellous, misleading, obscene or threatening material); - not use the website or app in a way which infringes upon the rights of third parties, such as, but not limited to, the rights of BrandNewDay and third parties with regard to privacy and intellectual property.

3. Intellectual property rights

All software, programs, databases, texts, advice, drawings, photos, films, images, data, names, brand and domain names, trademarks, logos, designs, information and other components of the website and app ("information") are protected by intellectual rights and belong to BrandNewDay or to third parties. It is forbidden to reproduce, alter, make public, distribute or send, sell, make available or otherwise transfer the information offered via the website and app (except for temporary reproduction of a transitory or incidental nature with a view to legitimate use and with no independent financial value) or grant any rights hereto to third parties without the prior written consent of BrandNewDay.

4. Liability of the user

In the event of non-compliance with or violation of intellectual rights or any other right of BrandNewDay or third parties (including the partners of BrandNewDay and other visitors to the website or app), you undertake to compensate, reimburse and indemnify BrandNewDay and the third parties in full for any claim, damage or liability of any kind whatsoever, including consequential damage, which may arise as a result of this.

5. Liability of BrandNewDay

5.1. Information on the website, app and via reporting

Information offered via the website, app or via reporting by BrandNewDay may be incomplete or contain errors. BrandNewDay does its best to work as accurately as possible, but is not responsible for the correctness, suitability, reliability, timeliness, exactness or any other errors in the information. The information is provided without any form of guarantee. BrandNewDay is not responsible for viruses or other information that could cause damage to the user or to third parties and accepts no liability whatsoever for any damage this may cause.

5.2. Advice on the website, app and via reporting

Advice or other forms of support offered via the website, app or via reporting by BrandNewDay, including that within the context of physical, mental, psychological, medical, legal, financial or other forms of support or service, may be incomplete or contain errors. BrandNewDay does its best to work as accurately as possible, but is not responsible for the correctness, suitability, reliability, timeliness, exactness or any other errors in such advice or other forms of support. The advice and other forms of support are provided without any form of guarantee. BrandNewDay is not responsible for advice and other forms of support that could cause damage to the user or to third parties and accepts no liability whatsoever for any damage this may cause. It is the responsibility of the user to always consult an expert for extra information and advice concerning the advice and information received by the user.

5.3. Links to other websites

The website and app may contain hyperlinks to websites or to third-party webpages or refer to these in some other way. BrandNewDay has no say over the contents of these websites or webpages and is not responsible for the content or characteristics thereof. The placing of links by BrandNewDay in no way signifies approval of the content of these websites or webpages.

5.4. Links to this website and app

The creation of hyperlinks to this website and app is only permitted provided these hyperlinks lead to the homepage of this website or the downloadpage of the app. Deep linking is not permitted without the prior written consent of BrandNewDay. The use of this website or parts thereof in other websites (for example, by automatically linking databases or other content via in-line linking, framing or in any other way) is expressly prohibited.

6. Privacy Policy

6.1. General

BrandNewDay is responsible for the processing of personal information within the meaning of the Act of 8 December 1992 on the protection of privacy with regard to the processing of personal information. Personal information is handled with care. For information on the privacy policy and the processing of personal information via the website and app, including exercising your right to inspection and correction, contact us via the information provided below.

6.2. Consent to process personal information.

By accessing and using the website or the app and the electronic messages of BrandNewDay, you are expressly agreeing to the content and terms and conditions of this privacy policy. When you visit the website or download the app, and when you provide BrandNewDay with personal information, you are giving BrandNewDay your express, written consent to gather, use and process the personal information in accordance with the purposes described below. You have the right to withdraw your consent at any time without specifying a reason.

6.3. What personal information is processed?

BrandNewDay processes the following details and personal information, among others. 6.3.1. Expressly provided information: the information expressly provided by you, such as user name, password, e-mail, sex, age, postal code and level of education, your choice to receive tips, your geolocation, etc. By providing this information, you are giving your express, written consent for it to be processed in accordance with the purposes described below. 6.3.2. Automatically gathered information: as well as the information gathered with your express, written consent, information may also be gathered automatically, such as your IP address, your computer's browser type, the operating system, your mobile device and other general information relating to your visit to the website or use of the app, such as generally measuring the number of visitors, the pages visited, downloads etc. 6.3.3. Cookies: during your visit, "cookies" may be placed on your computer's hard drive. Cookies help the website respond more efficiently to the wishes and preferences of our visitors. Accepting cookies is not compulsory. Access to the website is not dependent upon accepting cookies; at any time, you can use your browser's settings to stop cookies from being placed on your hard drive. In principle, your browser allows cookies to be blocked, warnings to be received before cookies are installed or installed cookies to be removed from your hard drive manually or automatically at the end of each browser session. See your Internet browser's instructions for details. 6.3.4. Sensitive information: if the processed information contains sensitive personal information, e.g. information about your lifestyle or psychosocial or medical information, then you are giving your express, written consent for the processing of the sensitive information in question via the procedure for signing up or registering new users and also from that point on for further processing, for the purposes described below.

6.4. Purposes of the processing

The personal information relating to you is processed with a view to allowing the website and app to function, analysing the visit and the processed information and sending newsletters. As a result of taking part in the programme, information is processed with a view to general support, including practical advice (customised tips to change your habits), diaries (recording habits and supporting information in a diary and monthly report), summary charts (to follow the evolution of habits), motivation assessments (to analyse and boost motivation), daily tips (sent via e-mail), mini-tests, a forum and with a view to processing anonymous information for scientific and statistical research purposes. Except with your express consent, personal information is not processed with a view to direct marketing. The information is also only processed by contractually-bound third parties, if necessary for the specified purposes, such as to programmers to allow the information technology to function, to service providers for hosting information, to researchers for processing in connection with statistical and scientific purposes, etc.

6.5. Right to access and improvements and changes to the privacy policy.

You have the right at all times to inspect and correct your personal information. You may submit a request to this effect via the information provided below. Any changes to the privacy policy will be announced via this page. You undertake to check this page regularly.

7. Applicable legislation and courts having jurisdiction

Belgian law applies to the website and app, with the exception of legal provisions of peremptory law to the contrary. In the event of a dispute, the courts of the district of Leuven have sole jurisdiction.

8. Miscellaneous

Copyright © 2009 Brand New Day NV. iCoach® is a registered logo. All programs, information, works, data and databases are protected by copyright. All rights are expressly reserved.

9. Company details

NV Brand New Day

Park 51 Site

Persilstraat 51C, bus 301

3020 Herent - Belgium

R.L.P. 0480.270.358

Tel.: +32.(0)16.22.88.93

E-mail: [info@brandnewday.eu](mailto:info@brandnewday.eu)
