# Supplementary material for: Use of the Smoking Cessation App Ex-Smokers iCoach and Associations With Smoking-Related Outcomes Over Time in a Large Sample of European Smokers: Retrospective Observational Study
Source: J Med Internet Res. 2023 Aug 22;25:e45223. doi: 10.2196/45223 (PMC10481207; doi:10.2196/45223)
Supplement: Multimedia Appendix 2 [file jmir_v25i1e45223_app2.docx]

## MULTIMEDIA APPENDIX 2

Table S1. Country of residence of all iCoach users.^a^

|  | TCS 2013^b^ | n (%) of all users  (n = 311,567) |
| --- | --- | --- |
|  |  |  |
| **Austria** |  |  |
|  | 31 | 2,603 (0.8) |
| **Belgium** |  |  |
|  | 47 | 12,904 (4.1) |
| **Bulgaria** |  |  |
|  | 46 | 12,936 (4.2) |
| **Cyprus** |  |  |
|  | 33 | 1,721 (0.6) |
| **Czech Republic** |  |  |
|  | 34 | 8,845 (2.8) |
| **Denmark** |  |  |
|  | 46 | 1,717 (0.6) |
| **Estonia** |  |  |
|  | 43 | 2,460 (0.8) |
| **Finland** |  |  |
|  | 55 | 1,792 (0.6) |
| **France** |  |  |
|  | 57 | 15,600 (5.0) |
| **Germany** |  |  |
|  | 32 | 12,217 (3.9) |
| **Greece** |  |  |
|  | 35 | 8,102 (2.6) |
| **Hungary** |  |  |
|  | 48 | 11,274 (3.6) |
| **Ireland** |  |  |
|  | 70 | 2,944 (0.9) |
| **Italy** |  |  |
|  | 46 | 18,261 (5.9) |
| **Latvia** |  |  |
|  | 41 | 2,048 (0.7) |
| **Lithuania** |  |  |
|  | 35 | 3,950 (1.3) |
| **Luxemburg** |  |  |
|  | 37 | 1,303 (0.4) |
| **Malta** |  |  |
|  | 56 | 1,614 (0.5) |
| **Netherlands** |  |  |
|  | 47 | 7,052 (2.3) |
| **Poland** |  |  |
|  | 43 | 16,921 (5.4) |
| **Portugal** |  |  |
|  | 41 | 12,802 (4.1) |
| **Romania** |  |  |
|  | 44 | 18,320 (5.9) |
| **Slovakia** |  |  |
|  | 39 | 6,882 (2.2) |
| **Slovenia** |  |  |
|  | 43 | 2,329 (0.7) |
| **Spain** |  |  |
|  | 56 | 28,170 (9.0) |
| **Sweden** |  |  |
|  | 48 | 2,295 (0.7) |
| **United Kingdom** |  |  |
|  | 74 | 94,505 (30.3) |

^a^ Numbers and frequencies are provided for all iCoach users in the present study.

^b^ Tobacco Control Scale 2013.

Table S2. App usage rate per duration of iCoach use.

| **Duration** (days)^1^ | 0-1 | 1-7 | 7-80 | 80-793 |
| --- | --- | --- | --- | --- |
| **N**^2^ | 207,131 | 44,173 | 30,642 | 29,621 |
| **Percentage**^3^ | 66.5% | 14.2% | 9.8% | 9.5% |
| **Mean rate** (min,max)^4^ | 1.13 (1.00, 21.00) | 0.774 (0.143, 32.00) | 0.258 (0.013, 7.125) | 0.069 (0.002, 6.413) |

^1^ duration of app usage in days.

^2^ number of iCoach users who used the app for this duration.

^3^ percentage of total number of iCoach users (n=311,567).

^4^ mean rate (including the minimum and maximum rate) of app usage for users who used to iCoach app for this duration.

Table S3. Baseline characteristics of all iCoach users.^a^

|  | | User groups^a^  (n = 26,785) | Non-user group^a^  (n = 284,782) | P | All users^a^  (n = 311,567) |
| --- | --- | --- | --- | --- | --- |
|  | |  |  |  |  |
| **Age** (years)^b^, mean (SD) | |  |  |  |  |
|  | | 36.5 (11.4) | 33.0 (10.5) | <.001 | 33.3 (10.6) |
| **Female**, n (%) | |  |  |  |  |
|  | | 12,695 (47.4) | 110,672 (38.9) | <.001^h^ | 123,367 (39.6) |
| **Education**, n (%) | |  |  |  |  |
|  | **Primary education** | 877 (3.3) | 19,434 (6.8) | <.001^h^ | 20,311 (6.5) |
|  | **Secondary education** | 9,802 (36.6) | 124,421 (43.7) |  | 134,223 (43.1) |
|  | **Higher education** | 16,106 (60.1) | 140,927 (49.5) |  | 157,033 (50.4) |
| **Professional Category**, n (%) | |  |  |  |  |
|  | **Employed** | 17,743 (66.2) | 172,922 (60.7) | <.001^h^ | 190,665 (61.2) |
|  | **Unemployed** | 2,004 (7.5) | 30,035 (10.5) |  | 32,039 (10.3) |
|  | **All other**^c^ | 7,038 (26.3) | 81,825 (28.7) |  | 88,863 (28.5) |
| **Country**^d^, n (%) | |  |  |  |  |
|  | **Belgium** | 2,151 (8.0) | 10,753 (3.8) | <.001^h^ | 12,904 (4.1) |
|  | **France** | 1,584 (5.9) | 14,016 (4.9) |  | 15,600 (5.0) |
|  | **Germany** | 1,285 (4.8) | 10,932 (3.8) |  | 12,217 (3.9) |
|  | **Hungary** | 1,419 (5.3) | 9,855 (3.5) |  | 11,274 (3.6) |
|  | **Italy** | 2,161 (8.1) | 16,100 (5.7) |  | 18,261 (5.9) |
|  | **Netherlands** | 1,138 (4.2) | 5,914 (2.1) |  | 7,052 (2.3) |
|  | **Poland** | 1,275 (4.8) | 15,646 (5.5) |  | 16,921 (5.4) |
|  | **Portugal** | 1,488 (5.6) | 11,314 (4.0) |  | 12,802 (4.1) |
|  | **Romania** | 1,187 (4.4) | 17,133 (6.0) |  | 18,320 (5.9) |
|  | **Spain** | 3,157 (11.8) | 25,013 (8.8) |  | 28,170 (9.0) |
|  | **United Kingdom** | 3,978 (14.9) | 90,527 (31.8) |  | 94,505 (30.3) |
| **TCS**, mean (SD)^e^ | |  |  |  |  |
|  | | 50.1 (12.4) | 54.5 (14.8) | <.001 | 54.2 (14.7) |
| **Smoking**, n (%) | |  |  |  |  |
|  | | 22,027 (82.2%) | 256,508 (90.1) | <.001^h^ | 278,535 (89.4) |
| **Quitting stage**, n (%) | |  |  |  |  |
|  | **Stage 1** | 467 (1.7) | 9,562 (3.4) | <.001^h^ | 10,029 (3.2) |
|  | **Stage 2** | 11,144 (41.6) | 142,953 (50.2) |  | 154,097 (49.5) |
|  | **Stage 3** | 10,416 (38.9) | 103,993 (36.5) |  | 114,409 (36.7) |
|  | **Stage 4** | 4,238 (15.8) | 21,122 (7.4) |  | 25,360 (8.1) |
|  | **Stage 5** | 520 (1.9) | 7,152 (2.5) |  | 7,672 (2.5) |
| **Cigarettes per day**^f^, mean (SD) | |  |  |  |  |
|  | | 16.5 (8.3) | 17.5 (8.6) |  | 17.4 (8.8) |
| **HSI**^g^, n (%) | |  |  |  |  |
|  | **Low (0-1)** | 5,398 (20.2) | 49,952 (17.5) | <.001^h^ | 55,350 (17.8) |
|  | **Medium (2-4)** | 13,938 (52.0) | 163,403 (57.4) |  | 177,341 (56.9) |
|  | **High (5-6)** | 2,771 (10.3) | 36,271 (12.7) |  | 39,042 (12,5) |
| **Motivation to stop**, mean (SD) | |  |  |  |  |
|  | | 4.1 (1.6) | 3.8 (1.8) | <.001 | 3.8 (1.8) |
| **Self-efficacy**, mean (SD) | |  |  |  |  |
|  | | 2.9 (1.9) | 2.8 (1.9) | <.001 | 2.8 (1.9) |
| **Importance of quitting**, mean (SD) | |  |  |  |  |
|  | | 5.3 (1.2) | 4.9 (1.5) | <.001 | 4.9 (1.5) |
| **Smoking in social environment**, mean (SD) | |  |  |  |  |
|  | | 2.5 (1.4) | 2.9 (1.5) | <.001 | 2.9 (1.5) |

^a^ User groups: users who were included in the trajectory analysis and were assigned to a groups based on usage pattern (Low, Mild, Moderate, Intensive); Non-user group: users not included in the trajectory analysis; All users: both user groups and the non-user group.

^b^ Range 18 – 93 years.

^c^ All other includes: retired, homemaker, student, other.

^d^ Listed countries with frequencies >4% within the user groups, see the Multimedia Appendix for the frequencies of all 27 countries.

^e^ Range 31 – 74.

^f^ Range 0 – 40, a total of > 40 cigarettes per day was computed as 40 cigarettes per day [58].

^g^ % of all users in each group; percentage of user missing overall: 12.8%.

^h^ The p value for the Pearson’s chi-squared test used to compare this categorical variable between the ‘user groups’ and the ‘non-user group’ is presented behind the first factor level of this variable.

P = P value for either the Pearson’s chi-squared test used to compare the distribution between categorical variables and the student t-test to compare means between continuous variables, between the ‘user groups’ and the ‘non-user group’. TCS = tobacco control scale, HSI = heaviness of smoking index.

Table S4. Descriptive statistics on total number of logins for iCoach usage groups per period.

| Period^a^ | Low^b^ | | Mild^b^ | | Moderate^b^ | | Intensive^b^ | |
| --- | --- | --- | --- | --- | --- | --- | --- | --- |
|  | Mean (SD) | Median [range] | Mean (SD) | Median [range] | Mean (SD) | Median [range] | Mean (SD) | Median [range] |
| Period 1 (0-60 days) | 2.0  (1.27) | 2 [1, 7] | 3.0  (1.09) | 3 [2, 6] | 10.3 (4.25) | 9 [5, 28] | 34.3 (13.64) | 31 [17, 106] |
| Period 2 (60-120 days) | 1.7 (0.99) | 1 [0, 6] | 3.0  (1.09) | 3 [2, 6] | 8.9 (3.66) | 8 [3, 25] | 29.3 (13.52) | 26 [10, 106] |
| Period 3 (120-180 days) | 0.6 (0.8) | 0 [0, 4] | 3.0  (1.09) | 3 [2, 6] | 4.8 (4.89) | 5 [0, 22] | 15.2 (17.94) | 11 [0, 106] |
| Period 4 (180-240 days) | 0.4 (0.54) | 0 [0, 2] | 2.9 (1.07) | 3 [1, 6] | 3.1 (4.33) | 0 [0, 21] | 10.1 (16.6) | 0 [0, 92] |
| Period 5 (240-300 days) | 0.2 (0.42) | 0 [0, 1] | 2.6 (1.29) | 2 [0, 6] | 2.1 (3.95) | 0 [0, 19] | 7.4 (14.96) | 0 [0, 92] |
| Period 6 (300-360 days) | 0.2 (0.39) | 0 [0, 1] | 2.1 (1.44) | 2 [0, 5] | 1.6 (3.51) | 0 [0, 16] | 5.8 (13.92) | 0 [0, 92] |

^a^ Period of app use, corresponding to the numbering of periods in Figure 2

^b^ iCoach usage groups

Table S5. Multinomial logistic regression results of characteristics associated with iCoach user group membership.^a^

|  | | Mild^b^ | | | Moderate^b^ | | | Intensive^b^ | | |
| --- | --- | --- | --- | --- | --- | --- | --- | --- | --- | --- |
|  | | OR | 95% CI | P | OR | 95% CI | P | OR | 95% CI | P |
|  | |  |  |  |  |  |  |  |  |  |
| **Age** | |  |  |  |  |  |  |  |  |  |
|  | | 1.01 | 1.01 – 1.02 | <.001 | 1.03 | 1.03 – 1.04 | <.001 | 1.06 | 1.05 – 1.07 | <.001 |
| **Gender**^c^ | |  |  |  |  |  |  |  |  |  |
|  | | 0.82 | 0.76 – 0.89 | <.001 | 0.71 | 0.66 – 0.77 | <.001 | 0.90 | 0.77 – 1.06 | 0.20 |
| **Education**^d^ | |  |  |  |  |  |  |  |  |  |
|  | **Primary school** | 0.73 | 0.58 – 0.92 | 0.007 | 0.85 | 0.68 – 1.05 | 0.13 | 1.15 | 0.78 – 1.68 | 0.48 |
|  | **Secondary school** | 0.98 | 0.91 – 1.06 | 0.64 | 1.02 | 0.95 – 1.11 | 0.59 | 1.20 | 1.02 – 1.41 | 0.03 |
| **Professional Category**^e^ | |  |  |  |  |  |  |  |  |  |
|  | **Employed** | 1.03 | 0.94 – 1.12 | 0.52 | 0.90 | 0.83 – 0.99 | 0.02 | 0.71 | 0.60 – 0.84 | <.001 |
|  | **Unemployed** | 0.97 | 0.83 – 1.14 | 0.71 | 0.93 | 0.80 – 1.09 | 0.37 | 0.84 | 0.61 – 1.14 | 0.26 |
| **HSI** | |  |  |  |  |  |  |  |  |  |
|  | | 0.97 | 0.94 – 0.99 | 0.006 | 0.93 | 0.90 – 0.95 | <.001 | 0.94 | 0.89 – 0.99 | 0.02 |
| **Self-efficacy** | |  |  |  |  |  |  |  |  |  |
|  | | 0.97 | 0.95 – 0.99 | 0.009 | 0.98 | 0.96 – 1.00 | 0.03 | 0.96 | 0.92 – 1.00 | 0.04 |
| **Importance of quitting** | |  |  |  |  |  |  |  |  |  |
|  | | 1.04 | 1.00 – 1.07 | 0.04 | 1.05 | 1.01 – 1.08 | 0.006 | 1.00 | 0.94 – 1.07 | 0.96 |
| **Smoking in social environment** | |  |  |  |  |  |  |  |  |  |
|  | | 0.90 | 0.87 – 0.93 | <.001 | 0.92 | 0.90 – 0.95 | <.001 | 0.91 | 0.86 – 0.97 | 0.004 |
| **TCS** | |  |  |  |  |  |  |  |  |  |
|  | | 0.98 | 0.98 – 0.99 | <.001 | 0.98 | 0.98 – 0.99 | <.001 | 0.98 | 0.97 – 0.98 | <.001 |

^a^ The analysis was performed with the four user groups.

^b^ The Low users are the reference group.

^c^ For Gender ‘females’ are the reference group.

^d^ For Education ‘higher education’ is the reference group.

^e^ For Professional Category the category ‘all other’ is the reference group (includes: retired, homemaker, student, other).

OR = Odd ratio, CI = 95% Confidence interval for the odds ratio, HSI = heaviness of smoking index, TCS = Tobacco Control Scale.

Table S6. Generalized estimating equations: prediction of smoking by iCoach user group over time.

|  | Estimate^a^ | SE | P |
| --- | --- | --- | --- |
|  |  |  |  |
| **Intercept** |  |  |  |
|  | 0.8107^b^ | 0.2191 | <.001 |
| **Mild**^c^ |  |  |  |
|  | 0.3419 | 0.1202 | 0.004 |
| **Moderate^c^** |  |  |  |
|  | 0.3151 | 0.1398 | 0.02 |
| **Intensive**^c^ |  |  |  |
|  | -0.0133 | 0.2097 | 0.95 |
| **Time 2** ^d^ |  |  |  |
|  | -1.1954 | 0.1056 | <.001 |
| **Time 3** ^d^ |  |  |  |
|  | -1.4043 | 0.1143 | <.001 |
| **Time 4**^d^ |  |  |  |
|  | -1.4815 | 0.1171 | <.001 |
| **Age** |  |  |  |
|  | 0.0156 | 0.0025 | <.001 |
| **Female gender** |  |  |  |
|  | 0.1212 | 0.0562 | 0.03 |
| **Secondary school** |  |  |  |
|  | -0.3316 | 0.1779 | 0.06 |
| **Higher education** |  |  |  |
|  | -0.3819 | 0.1745 | 0.03 |
| **Mild x Time 2** |  |  |  |
|  | -0.2804 | 0.1223 | 0.02 |
| **Moderate x Time 2** |  |  |  |
|  | -0.5495 | 0.1457 | <.001 |
| **Intensive x Time 2** |  |  |  |
|  | -0.1744 | 0.2225 | 0.43 |
| **Mild x Time 3** |  |  |  |
|  | -0.3175 | 0.1310 | 0.02 |
| **Moderate x Time 3** |  |  |  |
|  | -0.4527 | 0.1527 | 0.003 |
| **Intensive x Time 3** |  |  |  |
|  | -0.2434 | 0.2331 | 0.30 |
| **Mild x Time 4** |  |  |  |
|  | -0.3436 | 0.1345 | 0.01 |
| **Moderate x Time 4** |  |  |  |
|  | -0.3921 | 0.1546 | 0.01 |
| **Intensive x Time 4** |  |  |  |
|  | -0.4620 | 0.2344 | 0.049 |

^a^ Estimates are presented for smoking status. Smoking status was coded as: smoker = 1, non-smoker = 0.

b Intercept: estimate for Low users at the first time point.

c User groups; Low users are the reference group. Here the estimates are shown for the Mild, Moderate and Intensive users at Time 1.

d Time point; Time 1 = baseline HRA measurement, Time 2 = first follow-up HRA measurement at 3 months’ follow-up, Time 3 = second HRA measurement at 6 months’ follow-up, Time 4 = third HRA measurement at 9 months’ follow-up. Here the estimates are shown for time points for the Low users.

P = P value, SE = standard error.

Table S7. Generalized estimating equations: prediction of quitting stage by iCoach user group over time.

|  | Estimate^a^ | SE | P |
| --- | --- | --- | --- |
|  |  |  |  |
| **Intercept** |  |  |  |
|  | 2.95301^b^ | 0.09980 | <.001 |
| **Mild**^c^ |  |  |  |
|  | - 0.17822 | 0.04591 | <.001 |
| **Moderate**^c^ |  |  |  |
|  | - 0.18396 | 0.05057 | <.001 |
| **Intensive**^c^ |  |  |  |
|  | - 0.08737 | 0.08268 | 0.29 |
| **Time 2**^d^ |  |  |  |
|  | 0.51738 | 0.04131 | <.001 |
| **Time 3**^d^ |  |  |  |
|  | 0.75051 | 0.04846 | <.001 |
| **Time 4**^d^ |  |  |  |
|  | 0.89162 | 0.05282 | <.001 |
| **Age** |  |  |  |
|  | - 0.00399 | 0.00115 | <.001 |
| **Female gender** |  |  |  |
|  | - 0.05895 | 0.02573 | 0.02 |
| **Secondary school** |  |  |  |
|  | 0.13174 | 0.08251 | 0.11 |
| **Higher education** |  |  |  |
|  | 0.18925 | 0.08090 | 0.02 |
| **Mild x Time 2** |  |  |  |
|  | 0.09457 | 0.04666 | 0.04 |
| **Moderate x Time 2** |  |  |  |
|  | 0.16032 | 0.05343 | 0.003 |
| **Intensive x Time 2** |  |  |  |
|  | 0.06454 | 0.08942 | 0.47 |
| **Mild x Time 3** |  |  |  |
|  | 0.11386 | 0.05431 | 0.04 |
| **Moderate x Time 3** |  |  |  |
|  | 0.22274 | 0.06219 | <.001 |
| **Intensive x Time 3** |  |  |  |
|  | 0.08565 | 0.10167 | 0.40 |
| **Mild x Time 4** |  |  |  |
|  | 0.13960 | 0.06005 | 0.02 |
| **Moderate x Time 4** |  |  |  |
|  | 0.20775 | 0.06888 | 0.003 |
| **Intensive x Time 4** |  |  |  |
|  | 0.27788 | 0.10575 | 0.009 |

^a^ Estimates are presented for quitting stage on the original Likert-scale.

^b^ Intercept: estimate for Low users at the first time point.

^c^ User groups; Low users are the reference group. Here the estimates are shown for the Mild, Moderate and Intensive users at Time 1.

^d^ Time point: Time 1 = baseline HRA measurement, Time 2 = first follow-up HRA measurement at 3 months’ follow-up, Time 3 = second HRA measurement at 6 months’ follow-up, Time 4 = third HRA measurement at 9 months’ follow-up. Here the estimates are shown for time points for the Low users.

P = P value, SE = standard error.

Table S8. Generalized estimating equations: prediction of self-efficacy by iCoach user group over time.

|  | Estimate^a^ | SE | P |
| --- | --- | --- | --- |
|  |  |  |  |
| **Intercept** |  |  |  |
|  | 3.89322^b^ | 0.19437 | <.001 |
| **Mild**^c^ |  |  |  |
|  | - 0.33982 | 0.09819 | <.001 |
| **Moderate**^c^ |  |  |  |
|  | - 0.26764 | 0.11151 | 0.02 |
| **Intensive**^c^ |  |  |  |
|  | - 0.29912 | 0.17390 | 0.09 |
| **Time 2**^d^ |  |  |  |
|  | 0.62781 | 0.08947 | <.001 |
| **Time 3**^d^ |  |  |  |
|  | 0.93047 | 0.09656 | <.001 |
| **Time 4**^d^ |  |  |  |
|  | 1.06544 | 0.09331 | <.001 |
| **Age** |  |  |  |
|  | - 0.021484 | 0.00224 | <.001 |
| **Female gender** |  |  |  |
|  | - 0.28639 | 0.04987 | <.001 |
| **Secondary school** |  |  |  |
|  | 0.19571 | 0.15408 | 0.20 |
| **Higher education** |  |  |  |
|  | 0.29794 | 0.15094 | 0.048 |
| **Mild x Time 2** |  |  |  |
|  | 0.26777 | 0.10092 | 0.008 |
| **Moderate x Time 2** |  |  |  |
|  | 0.57601 | 0.11557 | <.001 |
| **Intensive x Time 2** |  |  |  |
|  | 0.75637 | 0.18050 | <.001 |
| **Mild x Time 3** |  |  |  |
|  | 0.24499 | 0.10855 | 0.02 |
| **Moderate x Time 3** |  |  |  |
|  | 0.41475 | 0.12428 | <.001 |
| **Intensive x Time 3** |  |  |  |
|  | 0.52716 | 0.19126 | 0.006 |
| **Mild x Time 4** |  |  |  |
|  | 0.27686 | 0.10567 | 0.009 |
| **Moderate x Time 4** |  |  |  |
|  | 0.46067 | 0.12155 | <.001 |
| **Intensive x Time 4** |  |  |  |
|  | 0.51648 | 0.17847 | 0.004 |

^a^ Estimates are presented for self-efficacy on the original Likert-scale.

^b^ Intercept: estimate for Low users at the first time point.

^c^ User groups; Low users are the reference group. Here the estimates are shown for the Mild, Moderate and Intensive users at Time 1.

^d^ Time point: Time 1 = baseline HRA measurement, Time 2 = first follow-up HRA measurement at 3 months’ follow-up, Time 3 = second HRA measurement at 6 months’ follow-up, Time 4 = third HRA measurement at 9 months’ follow-up. Here the estimates are shown for time points for the Low users.

P = P value, SE = standard error.
